# Changes in Postpartum Insurance Coverage in the US During the COVID-19 Pandemic

**DOI:** 10.1001/jamahealthforum.2022.0688

**Published:** 2022-04-22

**Authors:** Erica L. Eliason, Jamie R. Daw, Maria W. Steenland

**Affiliations:** 1 Department of Health Services, Policy & Practice, Brown University School of Public Health, Providence, Rhode Island; 2 Department of Health Policy & Management, Columbia University Mailman School of Public Health, New York, New York; 3 Population Studies and Training Center, Brown University, Providence, Rhode Island

## Abstract

This cross-sectional study examines changes in postpartum insurance churn during the COVID-19 pandemic.

## Introduction

Approximately 63% of maternal deaths in the US occur postpartum,^1^ yet nearly one-third of individuals experience postpartum insurance loss or changes (ie, churn).^2^ Medicaid-insured pregnant people are more likely to experience churn because pregnancy-related Medicaid ends after 60 days postpartum, and Medicaid eligibility for parents is more restrictive (on average, 40% of the federal poverty level [FPL] in nonexpansion states and 138% of the FPL in expansion states compared with a median 200% of the FPL during pregnancy).^3^ The COVID-19 pandemic potentially affected postpartum coverage through job losses and policy changes that increased private insurance subsidies and prevented Medicaid disenrollment, including after childbirth. This study examined changes in postpartum insurance churn during the COVID-19 pandemic.

## Methods

We used the 2019 to 2021 Current Population Survey, Annual Social and Economic Supplement (CPS-ASEC), and the annual sample size was approximately 98 000 households (Figure; eTable in the Supplement). We included female respondents aged 18 to 44 years who were living with a child younger than 1 year. Additional information on the data are available in the eMethods in the Supplement. This study was considered not human participants research by the Brown University institutional review board and followed the Strengthening the Reporting of Observational Studies in Epidemiology (STROBE) reporting guidelines for cross-sectional studies.

**Figure.**
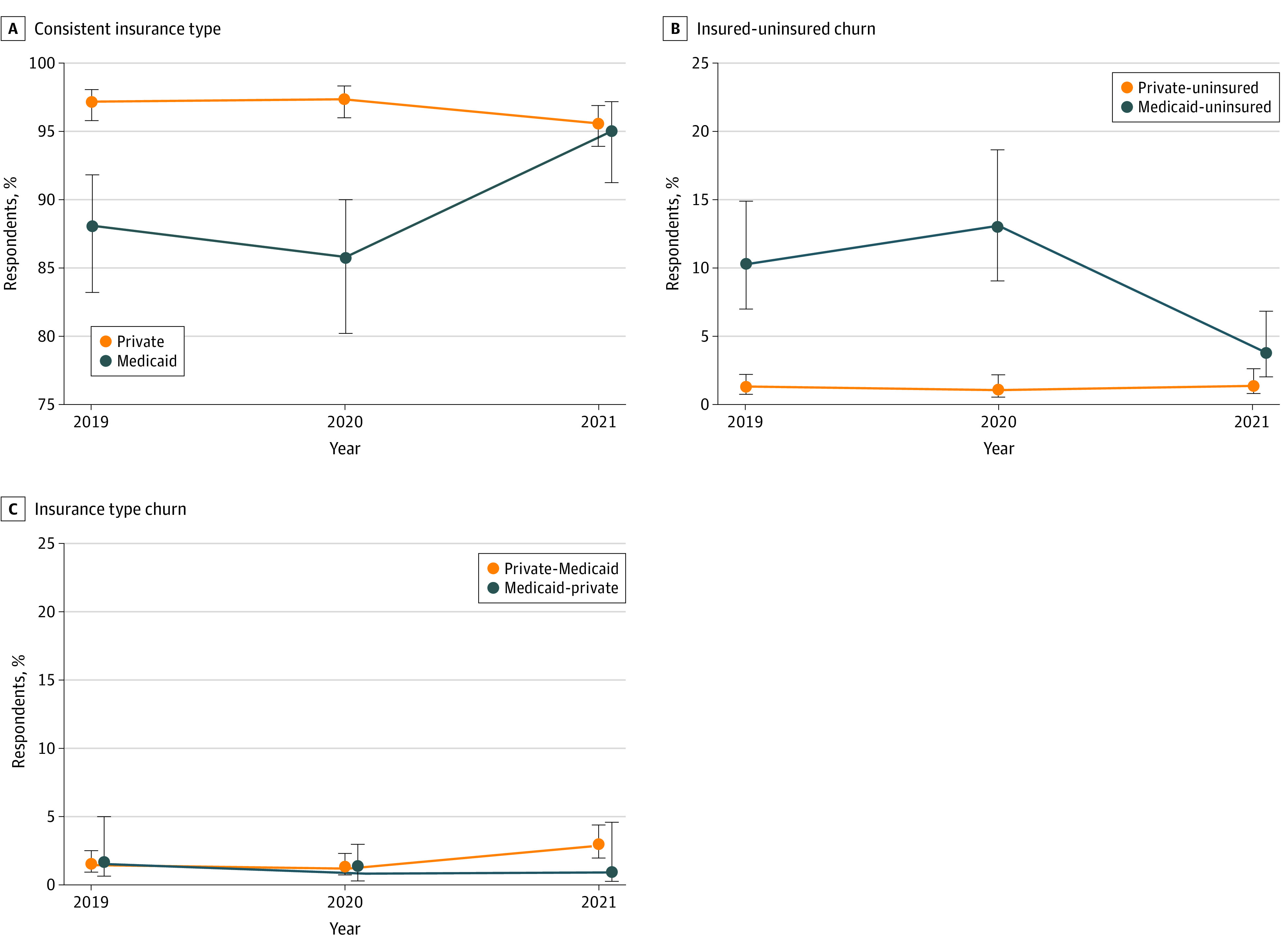
Unadjusted Trends in Postpartum Insurance Churn by Insurance Type Last Year From 2019 to 2021 Analysis of data from the Current Population Survey, Annual Social and Economic Supplement (CPS-ASEC). Sample includes females aged 18 to 44 years old living with a child of their own younger than 1 year. Data are weighted with CPS-ASEC survey weights. Confidence intervals for proportions are logit transformed. A total of 29 respondents were excluded because of missing insurance information.

Postpartum respondents reported coverage at (1) the time of the survey (ie, within 1 year postpartum) and (2) during the last calendar year (ie, the likely payer for pregnancy/birth). Coverage was assigned hierarchically to mutually exclusive categories: private, Medicaid, or uninsured. We defined churn as insurance loss or changes in insurance type between current insurance and insurance last year. We defined 5 patterns of coverage: (1) consistent insurance type (same insurance type last year and currently), (2) insurance type churn (change from Medicaid last year to current private, or vice versa), (3) insured-uninsured churn (any insurance last year to current uninsurance), (4) uninsured-insured churn (uninsurance last year to any current insurance), and (5) consistent uninsured (uninsured last year and currently). We examined postpartum insurance churn overall and by insurance type last year (Medicaid or private).

We defined 3 periods: prepandemic (2019), early pandemic (2020), and pandemic (2021). We used weighted regressions with year fixed effects, with an indicator for 2021 to identify differences in insurance churn rates in 2021 compared with 2019. Analyses were conducted using Stata, version 17 (StataCorp).

## Results

From 2019 to 2021, the CPS-ASEC response rate was 64.6%. Among all postpartum respondents (n = 4448), insured-uninsured churn decreased by 1.3 percentage points (95% CI, –2.5 to –0.0) from 3.1% in 2019 to 1.8% in 2021 (Table). No other coverage changes were observed overall.

**Table.** Changes in Postpartum Insurance Churn During the COVID-19 Pandemic From 2019 to 2021^a^

Outcomes:	% (95% CI)			
	Prepandemic (2019)	Early pandemic (2020)	Pandemic (2021)	Change in 2021 compared with 2019
Panel 1: all postpartum respondents (n = 4448, weighted N = 8 459 124)				
Consistent insurance type	89.5 (87.7 to 91.1)	88.2 (86.0 to 90.2)	87.8 (85.6 to 89.7)	–1.7 (–4.4 to 1.0)
Insurance type churn	1.3 (0.8 to 2.2)	1.0 (0.6 to 1.8)	2.2 (1.4 to 3.3)	0.8 (–0.3 to 1.9)
Insured-uninsured churn	3.1 (2.2 to 4.2)	3.6 (2.6 to 5.0)	1.8 (1.2 to 2.8)	–1.3 (–2.5 to –0.0)^b^
Uninsured-insured churn	0.5 (0.2 to 1.1)	0.2 (0.1 to 0.7)	0.9 (0.4 to 2.1)	0.4 (–0.5 to 1.3)
Consistent uninsured	5.4 (4.4 to 6.8)	6.7 (5.2 to 8.6)	7.2 (5.8 to 8.9)	1.7 (–0.2 to 3.7)
Panel 2: postpartum respondents with Medicaid during the last year (n = 940, weighted N = 1 796 673, weighted share = 21.2%)				
Consistent Medicaid	88.2 (83.2 to 91.8)	85.8 (80.2 to 90.0)	95.0 (91.3 to 97.2)	6.8 (1.7 to 11.9)^c^
Medicaid-uninsured	10.3 (7 to 14.9)	13.1 (9.1 to 18.7)	3.7 (2.0 to 6.9)	–6.6 (–11.1 to –2.0)^c^
Medicaid-private	1.6 (0.5 to 4.9)	0.7 (0.2 to 2.9)	0.8 (0.1 to 4.5)	–0.7 (–3.0 to 1.6)
Panel 3: postpartum respondents with private coverage in the last year (n = 3206, weighted N = 6 018 813, weighted share = 71.2%)				
Consistent private	97.1 (95.8 to 98)	97.4 (96.0 to 98.3)	95.6 (93.9 to 96.9)	–1.5 (–3.3 to 0.3)
Private-Medicaid	1.4 (0.8 to 2.4)	1.2 (0.6 to 2.3)	2.9 (1.9 to 4.3)	1.5 (0.0 to 2.9)^b^
Private-uninsured	1.3 (0.8 to 2.2)	1.1 (0.6 to 2.1)	1.4 (0.8 to 2.6)	0.1 (–1.0 to 1.2)

^a^ Authors’ analysis of data from the Current Population Survey, Annual Social and Economic Supplement (CPS-ASEC). Sample includes females aged 18 to 44 years old living with a child of their own younger than 1 year. Data are weighted with CPS-ASEC survey weights. Confidence intervals for proportions are logit transformed. A total of 29 respondents were excluded because of missing insurance information.

^b^ *P* < .05.

^c^ *P* < .01.

In 2019 (before the pandemic), among postpartum respondents with Medicaid during the last year, 88.2% had consistent Medicaid, 10.3% lost coverage, and 1.6% switched to private coverage. In 2021 (during the pandemic), consistent Medicaid increased by 6.8 percentage points (95% CI, 1.7 to 11.9) and Medicaid-uninsured churn decreased by 6.6 percentage points (95% CI, –11.1 to –2.0), representing a 64% decline from 2019.

In 2019, nearly all postpartum respondents (4319 [97.1%]) with private coverage during the last year maintained consistent private coverage, while 62 (1.4%) experienced private-Medicaid churn, and 58 (1.3%) lost coverage. Compared with 2019, private-Medicaid churn increased by 1.5 percentage points (95% CI, 0.0 to 2.9) in 2021.

## Discussion

Using nationally representative survey data, we found that postpartum insurance loss decreased during the pandemic, primarily associated with large increases in consistent Medicaid coverage. These findings suggest that the Families First Coronavirus Response Act, which prevented Medicaid disenrollment, was associated with substantial reductions in postpartum Medicaid loss. The finding of increased private-Medicaid churn is consistent with evidence that private coverage declines during the pandemic were largely offset by Medicaid increases.^4^

This study’s limitations include that the CPS-ASEC has historically undercounted people with Medicaid.^5^ Also, for early postpartum respondents, coverage during the past year may not necessarily reflect coverage during pregnancy or the 60 days postpartum because the past year covered preconception, pregnancy, and early postpartum. Currently, the Families First Coronavirus Response Act is set to expire in April 2022. Passage of legislation, which is under consideration in many states, to extend pregnancy-related Medicaid through 1 year postpartum is necessary to maintain pandemic-era gains in postpartum insurance continuity.^6^

## Notes

Supplement. **eTable. **Current Population Survey Annual Social and Economic Supplement Response Rates, 2019-2021 **eMethods. **Data, Sample Definition, and Insurance Variables
