# Supplementary material for: Changes in Postpartum Insurance Coverage in the US During the COVID-19 Pandemic
Source: JAMA Health Forum. 2022 Apr 22;3(4):e220688. doi: 10.1001/jamahealthforum.2022.0688 (PMC9034403; doi:10.1001/jamahealthforum.2022.0688)
Supplement: Supplement. — eTable. Current Population Survey Annual Social and Economic Supplement Response Rates, 2019-2021 eMethods. Data, Sample Definition, and Insurance Variables [file jamahealthforum-e220688-s001.pdf]

Supplemental Online Content

Eliason EL, Daw JR, Steenland MW. Changes in postpartum insurance coverage in the US during the COVID-19 pandemic. *JAMA Health Forum*. 2022;3(4):e220688. doi:10.1001/jamahealthforum.2022.0688

eTable. Current Population Survey Annual Social and Economic Supplement Response Rates, 2019-2021
eMethods. Data, Sample Definition, and Insurance Variables
eReferences

This supplemental material has been provided by the authors to give readers additional information about their work.

© 2022 Eliason EL et al. *JAMA Health Forum.*

eTable. Current Population Survey Annual Social and Economic Supplement Response Rates, 2019-2021

Survey Year	Unweighted Response Rate
2019	67.6
2020	61.1
2021	65.0
Mean, 2019-21	64.6

© 2022 Eliason EL et al. *JAMA Health Forum.*

eMethods. Data, Sample Definition, and Insurance Variables

Current Population Survey Annual Social and Economic Supplement Data:
Data collection for the Current Population Survey Annual Social and Economic Supplement (CPS-ASEC) occurs February-April each year, sampling one-quarter of the February and April CPS samples in addition to all March respondents. The CPS-ASEC also includes selected interviews with respondents from the preceding August-November CPS samples. The sample size is approximately 98,000 households per year.[1]

Because the majority of the CPS-ASEC data collection occurs in March, the 2020 data collection period was affected by the early COVID-19 pandemic. To protect the health of Census respondents and staff, after March 20th 2020, the Census switched from in-person interviews to telephone interviews.[2] As a result, as shown in eTable 1, the 2020 survey had lower response rates than prior years. The Census has also reported that respondents and nonrespondents in 2020 were more different from one another compared to three prior survey years.[2] While Census weights account for non-response, the higher rate of nonresponse and differential nature of nonresponse for key demographic variables such as income and education in 2020 may have reduced the effectiveness of these weights. For this reason, the Census suggests exercising caution when using the 2020 data.[2]

Further, though response rates rose in 2021 relative to the rate in 2020, they were still lower than pre-pandemic response rates, and respondents were still more likely to be higher income than non-respondents, a difference that had not been observed prior to the pandemic. Due to lingering differences in the characteristics of respondents and non-respondents, and the potential that non-response weights may not fully account for differential non-response, 2021 estimates should be interpreted with some caution. Despite this data limitation, we adopted the same approach as the Census[3] – comparing 2021 coverage to coverage in 2019 – to understand changes in insurance coverage during the pandemic.

Sample Definition:
The CPS-ASEC data does not include an indicator for whether a respondent had a birth in the past year. Thus, to identify postpartum respondents, we limited the study sample to female respondents aged 18-44 living with an infant of their own under age one. This approach was recently used by the U.S. Department of Health and Human Services[4] to study postpartum coverage with CPS-ASEC data. This definition has limitations. First, it will capture some respondents who are not biological parents because "own children" can include stepchildren and adopted children. However, the vast majority of identified respondents are likely to be biological parents with a recent birth. One study using American Community Survey (ACS) data, which includes a similar question about infants under the age of one in the household, found that 92% of respondents who reported an infant under the age of one in the household also reported a birth in the past year.[5] Second, this approach only captures individuals who identify as female. Third, since the child living with the respondents could be any age under one, we do not have information on the specific timing of the birth.

Statistical Methods:

© 2022 Eliason EL et al. *JAMA Health Forum.*

For each outcome, we estimated weighted linear probability models with year fixed-effects. The coefficient of interest was the indicator for survey year 2021. To compare 2021 and 2019 data without 2020 in the models, 2020 data was coded as missing, 2021 was coded as 1, and 2019 was coded as 0, representing the change in outcomes in 2021 relative to 2019.

Insurance Variables:
In the CPS-ASEC, insurance in the last year was asked "for any point during the previous year." For coverage in the last year and current coverage type, a respondent could report more than one insurance. In these instances, insurance was assigned hierarchically: private insurance included individuals with private alone or with other coverage, Medicaid insurance included individuals with Medicaid and no other coverage source, and uninsurance included only individuals with no coverage. Uninsurance in the last year was defined as having had no health insurance coverage at any time last year, i.e. those who were uninsured for the full last year.

Since we identify postpartum individuals based on whether they were living with an infant under age one, our sample of postpartum people could be anywhere between 0 to 11 months postpartum at the time of the CPS-ASEC survey. The amount of time in the respondent's one year lookback period for the survey that includes pregnancy or postpartum will vary based on when the infant was born relative to the survey date. Thus, for early postpartum respondents, coverage in the past year may not necessarily reflect coverage during pregnancy or during the 60 days postpartum because the past year covered pre-conception, pregnancy, and early postpartum.

eReferences

1. U.S. Census Bureau. Differences between the Current Population Survey (CPS) and the Annual Social and Economic Supplement (ASEC) to the CPS. Published October 8, 2021. https://www.census.gov/topics/population/foreign-born/guidance/cps-guidance/cps-vs-asec.html

2. U.S. Census Bureau. Current Population Survey 2021 Annual Social and Economic (ASEC) Supplement. Technical Documentation. Published March 2021. https://www2.census.gov/programs-surveys/cps/techdocs/cpsmar21.pdf

3. Keisler-Starkey K, Bunch LN. Health Insurance Coverage in the United States: 2020. United States Census Bureau. Published September 2021. Accessed February 17, 2022. https://www.census.gov/content/dam/Census/library/publications/2021/demo/p60-274.pdf

4. Gordon S, Sugar S, Chen L, Peters C, De Lew N, Sommers BD. Medicaid After Pregnancy: State-Level Implications of Extending Postpartum Coverage. Assistant Secretary for Planning and Evaluation (ASPE) Office of Health Policy Issue Brief. Published December 7, 2021. https://aspe.hhs.gov/sites/default/files/documents/cf9a715be16234b80054f14e9c9c0d13/medicaid-postpartum-coverage-ib%20.pdf

© 2022 Eliason EL et al. *JAMA Health Forum.*

5. Apostolova-Mihaylova M, Yelowitz A. Health Insurance, Fertility, And The Wantedness Of Pregnancies: Evidence From Massachusetts. *Contemp Econ Policy*. 2018;36(1):59-72. doi:10.1111/coep.12235

© 2022 Eliason EL et al. *JAMA Health Forum.*
